# The Institutional Library

**Published:** 1919-04-05

**Authors:** 


### THE INSTITUTIONAL LIBRARY.

### A MONUMENTAL MASTERPIECE.

### Dr. Norman Moore's History of Bart's.

The bulk of these two enormous volumes* is so great that, notwithstanding their quality, it is to be feared that they may adorn the shelves of more scholars than will read them. For, indeed, they cannot be held without the aid of mechanical appliances. The publisher frankly describes the two volumes as containing "1630 pages with 47 illustrations," the contents of which tell the history of the hospital from its foundation in 1123 to the present day." That great story is so well worth telling that it is a pity the author did not insist on distributing his pages between three or more smaller volumes. The generous margins and large type could have been sacrificed to smaller if more numerous sheets with advantage to the reader; although the splendour and clarity of the illustrations would have been much curtailed. Gibbon's "Decline and Fall," or Ruskin's "Modern Painters" would be unbearable in one volume, or even in two; and the latter work in its popular form shows how much can be done with reduced illustrations, even where, as in the instance of draughtsmanship or of cartulary texts, detail is of the first importance. A great work invariably entails the sacrifice of something. It is for the author to decide whether he will sacrifice the splendour of his pages or the convenience, and so the number, of his readers.

He begins by rightly pointing out that his work is a contribution to English history; because, as J. R. Green recognised (if somewhat timidly), the life of the English people through the centuries is really unfolded in the *grandeur et décadence* of their typical and favourite institutions. Unfortunately, Green made the mistake of acting as if he considered Parliament to be their typical and favourite institution. Really it is and was the largely distorting mirror of the methods and procedure of old town councils, monastic elections, and community guilds. The super-council of Parliament was and is their focal reflection, not the true or central type. The various forces governing the market life of the city were the real hub of the people's affairs. But Green *did* appreciate the importance of charters, town halls, shrines, and civic or municipal institutions. St. Bartholemew's Hospital in London was one of these healthy and vigorous city growths. Its history, as it actually occurred, therefore, is a history of England in the concrete; and its doings can only be understood, or indeed so much as recorded, if some part at least of varied and other historical matter be included. The scope of Dr. Moore's book is therefore fully justified. For a great institution attracts the friends of its chief, who becomes a man of prominence, and his contemporaries influence his policy just as his policy reacts upon the place. So Dr. Moore has given pen portraits of many of the hospitals' physicians, and surgeons throughout his story, and has tried also to record not only each man's place in the history of the profession, but also the effects of each man's work on the hospital where it was pursued. Each member of the great Pleiades—Rahere, Thomas of St. Osyth's, Alexander of Smithfield the Scribe, John Cok, the hospital's early recorder, Dr. John Caius, its quaint tenant, Harvey the Great, and John Abernethy, "the greatest teacher of his time"—each of these has a delightful chapter to himself. To that list must surely now be added the name of the hospital's most authoritative historian: whose preface ends with simple and affectionate words: "The history is a gift from me to St. Bartholomew's . . ." The frontispiece is a fine reproduction of what appears to be a fine portrait of the author by the artist Richard Grenville Eves. In the illustration, at all events, there is a Holbein quality, perhaps dictated by the fact that the sitter belongs physiognomically to the type which Holbein loved best to portray.

To review the whole would be to write another volume, historical and critical both. But such a review is fortunately impossible. Let us therefore taste the book and its story by a judicious mouthful here and there. The first shall be the words of S. Bartholomew wherein, in a vision, he encouraged Rahere to found the institution. We quote them because there can be no other encouragement for the beginners of any great task, whose lives are those but of trustees for its fulfilment. Do not, says the apostle, resolve yourself merely into a committee of ways and means, for "of this work, know that you shall be the servant, I the Lord; do you do the part of a servant, and I will discharge the duty of lord and patron." The final success of Rahere's efforts shows how practical this advice was. In answer to his petition, Henry I. granted him the site which he had desired, on which was eventually built the church of S. Bartholomew and the hospital house close by. His first appeals, let less enlightened persons please note, were not confined to personal solicitations, but included a series of sermons, though we are expressly told that these varied between "the sob and the smile," which the present chairman of the London Hospital has advocated. The first charter was granted to protect the founder from the plots of his opponents. Its date—1133—as the first of so many, deserves to be revered. The first miracle performed at the church was an orthopædic one, by which a cripple became able to walk. Alifumnus, whom Rahere chose to collect alms for the church and the poor patients, deserves to be remembered as the first financial secretary. Rahere himself died in 1145. The hospital, Dr. Moore points out, was built upon a gravel bed on the highest part of the open Smithfield site, exactly the spot which a modern architect would

* *The History of St. Bartholomew's Hospital.* By Norman Moore, M.D. 2 vols. Price £3 3s. net. London: C. Arthur Pearson, Ltd.

**The Institutional Library**—(*continued*).

choose. Though the Priory soon gained the precedence, it seems certain that the hospital was founded before the Priory and its church. Indeed, a hospital without a church would have been then as much of an anomaly as a hospital without a kitchen would be thought now. Nevertheless, the hospital's independence was confirmed by a series of charters, and this independence saved it at the Reformation from sharing the Priory's fate.

Without these grants in aid, it would have been impossible, in those times, to "carry on." The hospital church, said Henry in this charter, was "to be as free as my crown" and to be defended by the sovereign accordingly. They had also jurisdiction of "the rights of causes which may happen in their land." The foundations were also exempted from a multitude of duties. How heavy the burdens might be and how exasperating their pressure could become is known to those to whom hydages, averpane, muthbryche, schewight, forneng, and withfange are no mere mouthings, but grievous "forms" and obligations to be filled and met by those subject to Government Departments, Insurance Act officials, and the income tax accessor. The hospital early saw the desirability of being relieved from the depressing onus of D.O.R.A., and the bureaucracy of its day. To be relieved of Danegeld and utlepe to men of old time was, for their hospital successors of to-day, like being relieved from assessment to rates and death duties on legacies. The Augustinians being, like the modern Jesuits, more or less dispersed in active life, though yet obedient to a common rule, often had charge of hospitals.

Since this history is a history of the life and times of the people who founded S. Bartholomew's and maintained it, the legends recorded by the chroniclers and the miracles said to be worked at the church, and at Rahere's tomb have considerable importance. In no other way can the hopes and fears and daily avocations of Englishmen at the date in question appear. Without a record of miracles what should we know of pilgrimages, without a shrine to commemorate the miracle, would Gothic architecture itself have flourished? The very grotesques on misericords and corbels, now professedly admired, record the mind of the men who invented them. To admire the misericords and to scoff at the interest of the miracles is to prove our admiration of the very carving insincere.

The separate history of the hospital soon begins. As early as 1212 the hospital's relation to the mayors of London began. For London's first mayor, Henry Fitz-Ailwin, was its early benefactor, and how London came to have a mayor at all is one of the most interesting of Dr. Moore's delightful digressions, as is also the reference to the early handwriting of charters and correspondence. Only those whose hobby is printed books, or for whom Mr. Edward Johnson has made script a living subject, realise the interest and the beauty of penmanship, and how, with the invention of printing, the beauty of caligraphy began quickly to vanish away. For, as the voluntary hospital maintained the standard to which the State or municipal hospital seeks to conform, so did the pen maintain the standard of lettering, which the typefounder, when deprived of its example as a living art, allowed to lapse into decay. The argument for the voluntary system thus illustrated is obvious. Only a writer with his eye upon his object can be trusted not to digress too much, and it is because the thread of the tale is never lost that we wish to mention a few of the delicate strands out of which it is woven. Consequently, the examination of the charters yields more than a mere list of benefactions, and it is the reward of true scholarship to balance properly the minutiæ against the monuments of research. Thus, we are treated to the delightful picture of farm life in the thirteenth century which may be read on pages 239-244 from an early agreement. Remember, the hospital had to supervise the fetching of the hay from the farm, which involved the hiring of a ship and the provision of food for the sailors. We note that the lessee of the land was forbidden in the persons of himself and his heirs by the hospital procurator to "give, sell, or pledge the land to a Jew or a Jewess," or in any way to "alienate" the rent. The freedom of the river Fleet was given to the ships of the brethren, so far as right of way up and down it was concerned, by Robert, Warden of the Gaol of Fleet. And heavy barges may still be seen on the Thames with loads of Essex hay upon them. The river is now cribbed in an underground tunnel, as men were once in the upper prison associated with its name. But public opinion still runs through the dark tunnel of Fleet Street.

Henry III. gave an oak to the hospital: "The sick men at that time had their beds in a single hall, on the hearth of which the oaks were burned." In spite of the upheavals of his reign, the people bought and sold, trafficked and built, and the crafts flourished. Even Green himself makes this less clear than Dr. Moore. Louis's arrival in London was an incident, not an interruption. Business then had its artistic form in the exquisite penmanship of the scribes who wrote its documents. Of these Alexander of Smithfield wrote many, and, as Dr. Moore well says, "We may hope that such hands as wrote them touched the poor patients lying round the fire in the great hall." The deed in his script concerning the bookshop of William the Clerk is a beautiful piece of English. Whether this (I., p. 495) is a new translation is not indicated.

The charters themselves are not only invaluable for their beauty, but they have a humbler worth in that their clauses show the active working of Acts of Parliament, which then, as now, were often ignorantly drawn and required to be adapted, by the experience of those thereby affected, to the customs or the market, or the traditions of the shop. Into the later disputes between the Priory and the hospital, the Composition of Bishop Simon of Sudbury (1373) throws the light of actuality, and we learn not merely that there were quarrels, but how the men concerned in them felt and behaved, and of the way in which collectors for either institution were apt to steal each other's

**Institutional Library**—(*continued*).

thunder in their appeals. Simon was beheaded in 1381 by Wat Tyler's followers, and his head is still to be seen in the church of S. Gregory of Sudbury, in Norfolk. The trunk is buried at Canterbury. In 1381, Wat Tyler was killed; and the revolt which he led marks perhaps the most tremendous date in English history—for the turning-point then occurred from the old order to the new. Tyler was accommodated in the room of the hospital's master.

John Winfield's "Breviarium Bartholomei," "the first book on medicine connected with the hospital," conveniently closes the first volume and prepares us for the second, wherein the old order has changed, medicine became a modern science, accompts accounts, the staff slowly specialised, the architecture modern. On earlier ground, because it is less familiar, we have preferred to dwell, in the hope of showing what treasures lie in the remoter past, how hospital history is English history, and how the documents and charters are the historical tissue of national life, the very penmanship of which has alike its beauty and its lesson. St. Bartholomew's Hospital has received many splendid gifts, but we have no hesitation in saying that Dr. Moore's is the most magnificent of them.

**Sanitation in War.** By LIEUT.-COLONEL P. S. LELEAN, C.B., F.R.C.S., R.A.M.C. (London: J. and A. Churchill, 7 Great Marlborough Street. Price 7s. 6d.)

MESSRS CHURCHILL have brought out a third edition of Lieut.-Colonel Lelean's most interesting and instructive book, which was first published in 1915. Though primarily written for the use of medical officers and their assistants serving in the war, the value of the book is not decreased to any by the approach of peace, for it is full of information on all sorts of sanitary subjects, and will be useful to medical men living under advanced civilisation, and invaluable to those who seek their fortunes in the more primitive parts of the British Empire. The science of hygiene and the art of sanitation are dealt with broadly and tersely, yet with a wealth of detail that proves the author to be a master of condensation. A deal of most interesting information is given about the natural history of the many insects that are dangerous to man because they carry his diseases. The notes on marching are extremely interesting and practical, and will well repay study even by him who only contemplates a holiday walking tour. In fact the book is not only a book for medical men and sanitary orderlies; it is a book for every officer who has to handle men and be responsible for their health and welfare. It is a book for every intelligent citizen who wishes to know something of the preservation of health and of what it means to be at war or cantoned in the outposts of the Empire.

Sanitary science has made great studies in the last twenty-five year, and no body of medical men has done more for progress in it than the R.A.M.C. This book of Lieut.-Colonel Lelean's is no mean contribution to our knowledge, and is written in a style easily understood by those who have not special knowledge of medical science and hygiene. It deserves to be studied by a large portion of the public.

**The Science and Art of Deep Breathing.** By SHOZABURO OTABE M.D., Bâle, Assistant Medical Officer, Kensington Infirmary. Pp. viii + 114, with two illustrations and two plates. (London: Bale, Sons & Danielsson, 1919.).

RESPIRATION being the busiest physiological function which is partly voluntary has caused it to be a favourite with health culturists. Dr. Otabe, who is a superior specimen of this kind of enthusiast, maintains in this booklet that deep breathing for ten minutes morning and evening is a sure prophylactic of consumption, and a good means of treatment for a variety of disorders. He does not give himself the space to notice possible objections to his theory, so if we do it for him it must be in no carping spirit. Briefly, then, Dr. Haldane, the leading British authority on respiratory exchange, condemns breathing exercises as useless because there is a natural regulation of the amount of oxygen inhaled depending on that of the $CO_2$ in the blood. Again, the author thinks that to improve the lymph and blood circulation in the pulmonary apices will tend to cure tuberculosis deposits there. But a complete artificial pneumothorax, the remedial value of which is in certain cases undoubted, abolishes the one and lessens the other. Also he states that "active gaseous exchange hinders the development of the tubercle bacilli": but tubercle bacilli multiply better in the sputum than in the lung, it is often said, on account of readier access to oxygen. We note too his relation of an "interesting story" of a Japanese doctor who cured himself of consumption of eight years' standing by singing a song! However, nurses who are keen enough to manage a regular ten minutes for breathing exercises before going on duty in the morning may like to try Dr. Otabe's preventive all the same.

**A Manual of Chemistry.** By ARTHUR P. LUFF, M.D., F.R.C.P., and HUGH C. CANDY, B.A., B.Sc. Sixth Edition. (London: Cassell and Co., Ltd. 1918. Pp. 745 + xix. Price 12s. net.)

WE have had the pleasure of reviewing earlier editions of this book, and our favourable opinion of it may be regarded as confirmed by the judgment of the constituency to which it specially appeals. It has now arrived at a sixth edition, and this is practical testimony not needing an interpreter. A manual of chemistry for medical students is no light task to undertake. Clearly the science cannot be discussed in all its aspects and relations, for this would invade the curriculum at the expense of subjects more important from the medical standpoint. On the other hand, chemistry, it will be allowed, provides a most admirable mental discipline in the culture of scientific method, and any attempt to scamp it or to cram it must mean a definite loss in the training of the student. Still more, it is not difficult to believe that chemistry is going to play an increasingly prominent part in our knowledge of the nature of disease-processes, and those who would follow such a development must have the foundations well and truly laid. Thus the author of a student's text-book stands between two fires. If he overloads his book the student cannot find time for it, while if he cuts his explanations his educational influence will be restricted. Clearly, he must select his matter from the mass which is available, and he must keep principles, as distinct from details, well to the forefront. Still again, he will, so far as this is possible, illustrate his teaching by the consideration of materials which are likely to be familiar and helpful to his readers. The authors of the volume now before us have been guided by some such influences as those just sketched, and it is largely their faithfulness to these principles which have led to the success of their book. The student has appreciated the recognition of his peculiar needs and has expressed his gratitude in a convincing fashion. The success has been well merited, and it has therefore a double value.

